# Two-in-One Nanoparticle Formulation to Deliver a Tyrosine Kinase Inhibitor and microRNA for Targeting Metabolic Reprogramming and Mitochondrial Dysfunction in Gastric Cancer

**DOI:** 10.3390/pharmaceutics14091759

**Published:** 2022-08-23

**Authors:** Yu-Li Lo, Tse-Yuan Wang, Chun-Jung Chen, Yih-Hsin Chang, Anya Maan-Yuh Lin

**Affiliations:** 1 Institute of Pharmacology, National Yang Ming Chiao Tung University, Taipei 112, Taiwan; 2 Faculty of Pharmacy, National Yang Ming Chiao Tung University, Taipei 112, Taiwan; 3 Department of Medical Research, Taichung Veterans General Hospital, Taichung 407, Taiwan; 4 Department of Medical Laboratory Science and Biotechnology, China Medical University, Taichung 404, Taiwan; 5 Department of Biotechnology and Laboratory Science in Medicine, National Yang Ming Chiao Tung University, Taipei 112, Taiwan; 6 Department of Medical Research, Taipei Veterans General Hospital, Taipei 112, Taiwan

**Keywords:** nanoparticle, tyrosine kinase inhibitor, microRNA, mitochondrial targeting, tumor metabolism reprogramming, mitochondrial dysfunction

## Abstract

Dysregulational EGFR, KRAS, and mTOR pathways cause metabolic reprogramming, leading to progression of gastric cancer. Afatinib (Afa) is a broad-spectrum tyrosine kinase inhibitor that reduces cancer growth by blocking the EGFR family. MicroRNA 125 (miR-125) reportedly diminishes EGFRs, glycolysis, and anti-apoptosis. Here, a one-shot formulation of miR-125 and Afa was presented for the first time. The formulation comprised solid lipid nanoparticles modified with mitochondrial targeting peptide and EGFR-directed ligand to suppress pan-ErbB-facilitated epithelial–mesenchymal transition and mTOR-mediated metabolism discoordination of glycolysis–glutaminolysis–lipids. Results showed that this cotreatment modulated numerous critical proteins, such as EGFR/HER2/HER3, Kras/ERK/Vimentin, and mTOR/HIF1-α/HK2/LDHA pathways of gastric adenocarcinoma AGS cells. The combinatorial therapy suppressed glutaminolysis, glycolysis, mitochondrial oxidative phosphorylation, and fatty acid synthesis. The cotreatment also notably decreased the levels of lactate, acetyl-CoA, and ATP. The active involvement of mitophagy supported the direction of promoting the apoptosis of AGS cells, which subsequently caused the breakdown of tumor-cell homeostasis and death. In vivo findings in AGS-bearing mice confirmed the superiority of the anti-tumor efficacy and safety of this combination nanomedicine over other formulations. This one-shot formulation disturbed the metabolic reprogramming; alleviated the “Warburg effect” of tumors; interrupted the supply of fatty acid, cholesterol, and triglyceride; and exacerbated the energy depletion in the tumor microenvironment, thereby inhibiting tumor proliferation and aggressiveness. Collectively, the results showed that the two-in-one nanoparticle formulation of miR-125 and Afa was a breakthrough in simplifying drug preparation and administration, as well as effectively inhibiting tumor progression through the versatile targeting of pan-ErbB- and mTOR-mediated mitochondrial dysfunction and dysregulated metabolism.

## 1. Introduction

Gastric cancer (GC) is the fifth most common neoplasm, and it remains the third leading cause of cancer-related deaths globally [1]. The upregulation of human epidermal growth factor receptor family (HERs, EGFRs, or ErbBs), including HER1 (EGFR and ErbB-1), HER2, and HER3, has occurred in most GC cases [2,3]. HER2, which is found in approximately 15–20% of gastric adenocarcinomas, is frequently connected with locally advanced or metastatic status, which decreases the total survival of patients [4]. At present, although trastuzumab is an officially approved anti-HER2 monoclonal antibody (mAb) for HER2-overexpressed GC, resistance to this target therapy of HER family frequently occurs [4]. Dysfunctional EGFR, KRAS, and mammalian/mechanistic target of rapamycin (mTOR) pathway also cause metabolic reprogramming, leading to the progression of numerous cancers, including GC [5]. Furthermore, the co-amplification of HER2 and KRAS and the activation of mTOR pathway are positively correlated with poor prognosis and increasing resistance in GC treatment [2,6]. Genetic mutations in EGFR and phosphatidylinositol 3-kinase (PI3K)-protein kinase B (Akt) are frequently related to mTOR overexpression [7]. Therefore, effective multitargeting suppression of EGFR/HER2/HER3 by using pan-HER inhibitors may be a potential GC therapeutic approach. The dysregulational mitochondrial function, oxidative stress, PI3K/AKT/mTOR, and AMP-activated protein kinase (AMPK) signaling pathways all play important roles in metabolism reprogramming in tumor cells [8]. These changes in mitochondrial dynamics may affect the regulation of bioenergetics and the cellular oxidation-reduction (redox) equilibrium in cancer cells [9]. Thus, identifying a suitable tyrosine kinase inhibitor (TKI) with multiple functions in modulating the EGFR, KRAS, and mTOR pathways is important to reprogram tumor metabolism and reverse mitochondrial dysfunction.

Afatinib (BIBW2992; abbreviated as Afa), an oral pan-ErbB family inhibitor, is a potent second-generation EGFR-TKI [10]. It is used in patients with EGFR-mutated non-small cell lung cancer, and it displays clinical activity in HER2-positive breast cancer and GC [11,12]. Afa irreversibly binds to pan-ErbB tyrosine kinases, such as EGFR, HER2, and HER3, and, thus, exhibits anti-tumor efficacy by inducing cancer-cell apoptosis [10]. Furthermore, Afa effectively decreases the proliferation of GC cells with high EGFR/HER2 overexpression by selectively inhibiting the ErbB family and downstream mTOR and MAPK pathways [5]. The combined treatment of pan-HER, such as Afa, and PI3K inhibitors shows synergistic long-term regression against cervical tumors with aberrant HER2 and PI3K/AKT/mTOR pathway [2]. Although Afa is a potential EGFR-TKI for possible treatment of GC resistant to anti-EGFR mAb, Afa and EGFR form an irreversible covalent bond that produces painful side effects, such as rash/acne and diarrhea. Afa also has high lipophilicity and low aqueous solubility. Consequently, its sustained release at the site of tumors and the development of well-designed nanoparticles with dual functions of directing to the cancer target may minimize uncomfortable adverse events and, thus, provide a potential delivery strategy.

MicroRNA 125 (miR-125), comprising 22 nucleotides, is usually downregulated in numerous cancer types, including cervical cancer, GC, and breast cancer [13,14]. However, miR-125 is also changed in non-cancerous illnesses, such as maternal syndromes, highlighting the multi-faceted actions of this miRNA [15]. Interestingly, miR-125 expression levels are considerably downregulated in gastric adenocarcinoma with HER2-positive status [16]. MiR-125 is one of three miRNA signatures positively correlated with the survival of patients with GC by modulating the PI3K and mTOR signaling pathways [17]. Remarkably, miR-125 plays an important role as a tumor suppressor by regulating PI3K/Akt/mTOR, NF-κB, p53, HER2, and ß-catenin, thereby modulating cell growth/apoptosis, metabolism, resistance, and tumor immunity in various cancers, including GC [18]. It also participates in inhibiting glycolysis by downregulating hexokinase 2 (HK2), suppressing lipid metabolism, modulating respiratory chain to decrease oxygen consumption rate, regulating fusion and fission, and downregulating Bcl2 to induce apoptosis [19,20]. All these studies suggest the potential of miR-125 as a therapeutic target in EGFR- and/or HER2-positive tumor types, such as GC. However, the instability of miR during systemic circulation and the low transfection efficiency of naked miR into cells warrant the development of suitable systems for miR delivery [21]. Accordingly, the present study aimed to exploit the modulation of tumor-mitochondrion energy metabolism by affecting the EGFR/HERs/PI3K/AKT/mTOR pathway with miR-125 as an adjuvant therapy combined with Afa, a potent EGFR-TKI-targeting the broad-spectrum ErbB family, in a prospective nanoparticle formulation for potential GC therapy.

Solid lipid nanoparticles (SLNs), comprising amphiphilic lipids and surfactants, display benefits of tailored surface, high drug loading, good stability, and controlled release [22]. Anionic miR interacts with cationic lipids to form an electrostatic complex, and Afa may be stabilized by the mixture of lipids and surfactants. Thus, SLN is a suitable formulation for the intracellular delivery of Afa and miR-125. In particular, these SLNs are further altered by EGFR-targeted L peptide and mitochondrion-directed K peptide to increase targeting of Afa and/or miR towards GC cells and, thus, activate mitochondrion-mediated GC cell death.

**Figure 1:**
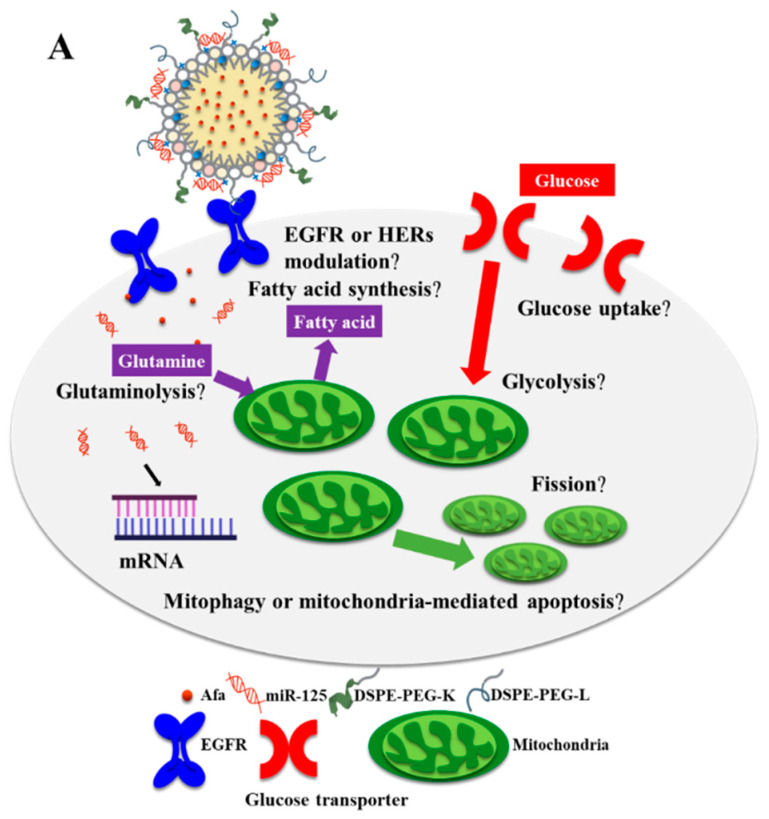

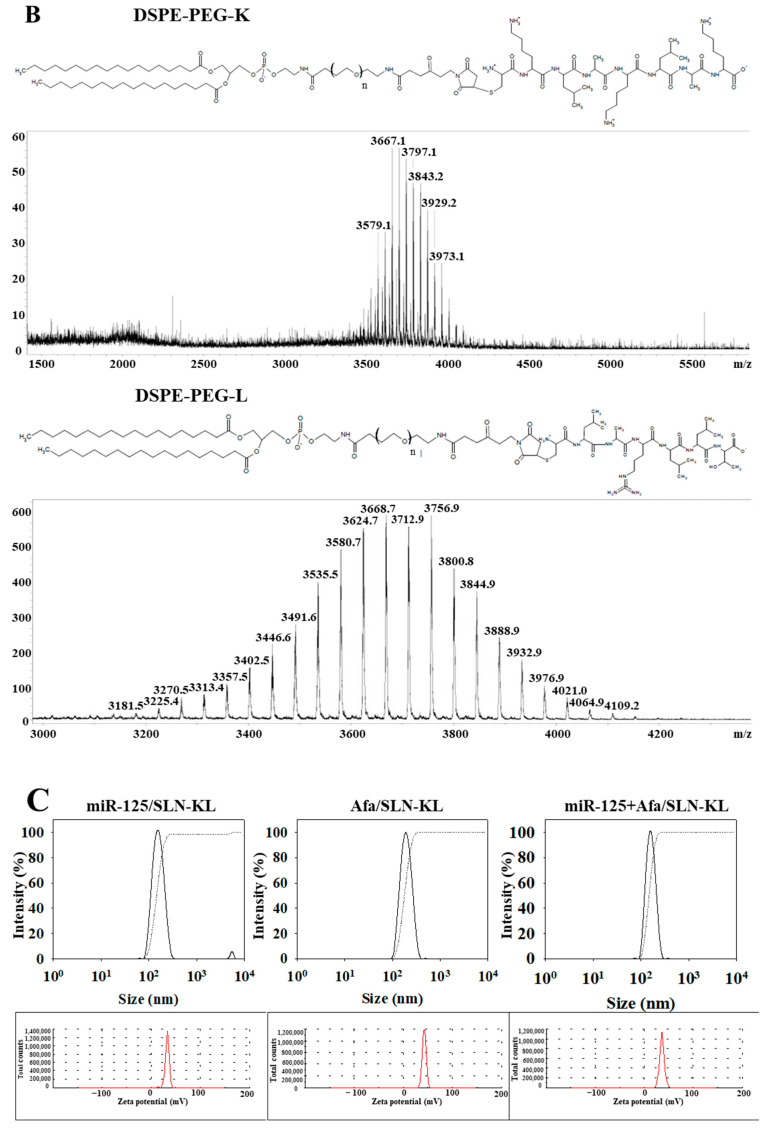

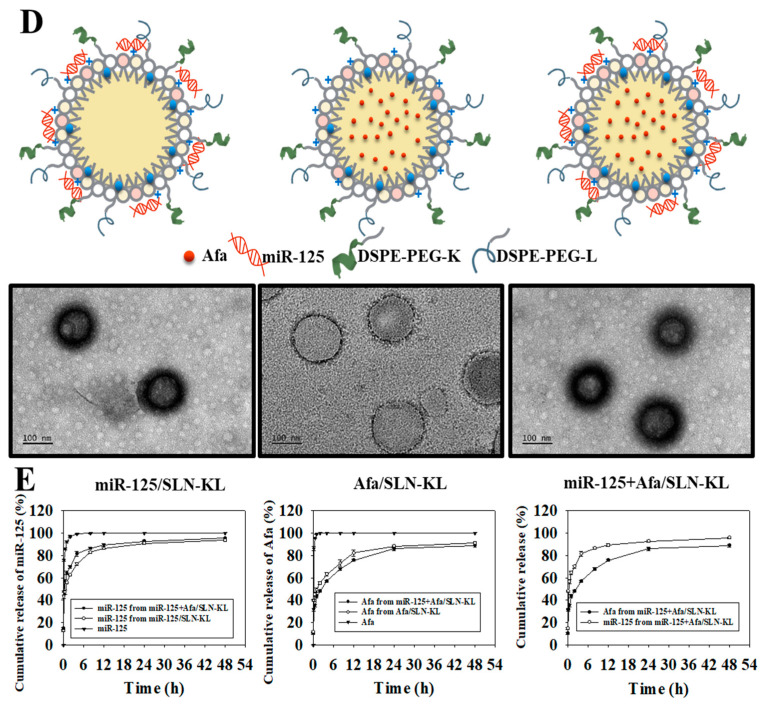
Rationale and physicochemical characterization of different miR-125 and/or Afa formulations. (**A**) Rationale of reprogramming metabolic dysregulation and dysfunctional mitochondria in AGS cells by miR-125 + Afa/SLN-KL. (**B**) Conjugation of DSPE-PEG to (**up**) K peptide and (**down**) L peptide. (**C**) Sizes and zeta potential of (**left**) miR-125/SLN-KL, (**middle**) Afa/SLN-KL, and (**right**) miR-125 + Afa/SLN-KL, as measured by Malvern Zetasizer. (**D**) TEM images of (**left**) miR-125/SLN-KL, (**middle**) Afa/SLN-KL, and (**right**) miR-125 + Afa/SLN-KL, as observed using JEM-2000EXII TEM. Scale bar, 100 nm. (**E**) In vitro release profiles of miR-125 and/or Afa from SLN-KL.

Mitochondrion-directed K peptide is a positively charged mitochondrion-targeting peptide. It exhibits electrostatic and hydrogen-bonding interactions between the cationic lysine group and anionic tumor-cell surface, thereby promoting the cellular uptake of nanoparticles into cancer cells [23]. The cationic domain of this proapoptotic K peptide reportedly binds and disturbs the anionic membranes of the mitochondria and subsequently increases cytotoxicity against different cancer cells [24]. EGFR-targeted L peptide is screened from a virtual peptide library by computer-assisted design [25]. L peptide binds specifically to EGFR in human colorectal cancer (CRC) and head and neck cancer cells [23,26]. Bioconjugates bearing L peptide also display localization in EGFR-overexpressed CRC cells [26]. The authors’ previous study has validated the EGFR-binding and tumor-targeting effects of linear L peptide by confocal laser scanning microscopy (CLSM) and flow cytometry [23]. The nanoparticles modified with L peptide may enter cancer cells via endocytosis after binding of L peptide to EGFR, thus enhancing tumor targeting and penetration [23,26]. Therefore, the tumor targeting and penetration of these tailored nanoparticles, particularly miR-125 + Afa/SLN-KL, in GC cells were increased by EGFR-directed L peptide and the subsequent ligand-mediated endocytosis. After escaping from endosomes and lysosomes, the released miR-125 and Afa were localized in the cytoplasm and mitochondria with the assistance of K peptide. The design of Afa + miR-125/SLN-KL is displayed in Figure 1A.

## 2. Materials and Methods

### 2.1. Materials

FAM-miR-125 and miR-125 were bought from GenePharma (Shanghai, China). Afa was purchased from MedChem (Monmouth Junction, NJ, USA). K and L peptides were synthesized by Kelowna (Taiwan). Cholesterol, L-α-phosphatidylcholine (PC), DOTAP, and Tween 80 were obtained from Acros (Geel, Antwerp, Belgium) and Avanti (Alabaster, AL, USA). All cell culture media and reagents were bought from Gibco BRL (Grand Island, NY, USA). Most of the other chemical reagents were purchased from either Merck (Darmstadt, Germany), Cayman (Ann Arbor, MI, USA), or Sigma-Aldrich (St. Louis, MO, USA).

### 2.2. Synthesis of Lipid-Peptide Conjugates (DSPE-PEG-K and DSPE-PEG-L)

DSPE-PEG-NHS and K peptide or L peptide were mixed together at a molar ratio 1:1 and the mixture was reacted overnight at room temperature in the dark. After dialysis using a 5 kDa cut-off membrane against phosphate-buffered saline (PBS) for 24 h, the lipid-peptide conjugates were lyophilized and stored at −20 °C.

### 2.3. Preparation of Peptide-Conjugated SLN Loaded with Single or Dual Drugs (miR-125/SLN-KL, Afa/SLN-KL, or miR-125 + Afa/SLN-KL)

PC, cholesterol, DOTAP, and DSPE-PEG-K or DSPE-PEG-L were mixed together in ethanol for 30 min at 50 °C. Then, 0.1% Tween 80 was added to the lipid dispersion and stirred for 30 min. Afterward, miR-125 was added to prepare miR-125/SLN-KL.

PC, cholesterol, DOTAP, and Afa were mixed together in ethanol for 30 min at 50 °C. Then, 0.1% Tween 80 was added to the lipid solution and stirred for 30 min to prepare Afa/SLN-KL. Furthermore, miR-125 was loaded to prepare miR-125 + Afa/SLN-KL.

### 2.4. Evaluation of Particle Size, Zeta Potential, and Morphology of Nanoparticles

The zeta potential, size distribution, and polydispersity index (PDI) of miR-125/SLN-KL, Afa/SLN-KL, and miR-125 + Afa/SLN-KL were studied by Zetasizer Nano-ZS particle size analyzer (Malvern Instruments Ltd., Malvern, Worcestershire UK). Each sample was measured at least three times. For morphology observation, the nanoparticle formulations were dipped on a carbon-coated copper grid and incubated for 1 min. These samples were stained by 2% uranyl acetate, incubated for 1 min, and examined under transmission electron microscope (TEM; JEM-2000EXII, Tokyo, Japan).

### 2.5. Encapsulation Efficiency (EE%) and Drug Loading Capacity (DL%)

A dispersion of miR-125 or Afa-loaded formulations was separated by ultracentrifuge at 15,000 rpm at 4 °C. Then, the harvested nanoparticles were incubated with 0.5% Triton X 100 for 30 min. miR-125 or Afa in the filtrate and broken nanoparticles were measured by NanoDrop (Thermo Fisher, Waltham, MA, USA) and UV/VIS Spectrophotometer at 260 and 450 nm (Ultrospec 8000 PC; Biochrom, Holliston, MA, USA), respectively. Each sample was analyzed in triplicate. EE% or DL% of miR-125, Afa, miR-125 + Afa in SLN-KL were computed by the following formula.

EE% = [(W_e_ − W_f_)/W_e_] × 100% (1)

DL% = [(W_e_ − W_f_)/W_t_] × 100% (2)

where W_e_ is the weight of added miR-125 or Afa, W_f_ is the weight of miR-125 or Afa in the filtrate, and Wt is the total nanoparticle weight.

### 2.6. Drug Release Study

The nanoparticles were incubated with PBS (pH 7.4) for the indicated time intervals (10 min, 30 min, 1, 2, 4, 8, 12, 24, and 48 h) at 37 °C. The concentration of miR-125 or Afa was detected by NanoDrop and UV/VIS Spectrophotometer, separately.

### 2.7. Cell Lines

AGS cells, a human gastric adenocarcinoma cell line from the metastatic site, were cultured in Roswell Park Memorial Institute (RPMI) 1640 medium accompanied with 10% fetal bovine serum (FBS) and 1% penicillin/streptomycin. Rat small intestine epithelial IEC-6 cells were cultured in Dulbecco’s modified Eagle’s medium (DMEM) supplemented with 10% FBS and 1% penicillin/streptomycin.

### 2.8. Cell Viability by SRB Assay and the Calculation of Combination Index (CI)

AGS and IEC-6 (8 × 10^3^) cells were seeded in 96-well plates overnight. Cells were treated with different miR-125 (100 nM) and/or Afa (300 nM)-loaded formulations. After 48 hours of incubation, cells were mixed with 1% TCA at 4 °C for 1 h. Then, they were stained with 0.04% sulforhodamine B (SRB) for 10 min. After air-drying at room temperature overnight, the plates were read on an ELISA reader (TECAN, Männedorf, Switzerland) at the absorbance of 540 nm.

To test the synergistic effect, the cells were treated with miR-125, Afa, and miR-125 + Afa in different concentrations. The CI was calculated by CompuSyn software (Paramus, NJ, USA) to assess the degree of drug interactions, where CI > 1, = 1, and < 1 indicate antagonistic, additive, and synergistic effect, respectively.

### 2.9. Cellular Uptake and Intracellular Localization of miR-125 and Afa in AGS Cells

AGS (7 × 10^4^) cells were seeded in 24-well plates. Cells were treated with FAM-miR-125 (100 nM) or DiI-Afa (100 ng/mL) encapsulated in SLN formulations. After 24 h of incubation, cells were collected and re-suspended into PBS in the dark. The fluorescence intensity of FAM-miR-125 or DiI-Afa accumulated in the cells was quantified using a FACSCalibur™ flow cytometer (Becton Dickinson, San Jose, CA, USA).

For intracellular localization of nanoparticle formulations, mitochondria were stained with MitoTracker^®^ Green or MitoTracker^®^ Red for 30 min. The cells were fixed in 4% paraformaldehyde at 37 °C for 10 min in the dark. After FBS-blocking, the cells were labeled with primary antibody against EGFR (Cell Signaling, Beverly, MA, USA) at 4 °C overnight, then labeled with secondary antibody of Cy5-AffiniPure Goat Anti-Rabbit Immunoglobulin G (Ig G; Jackson, PA, USA) at 4 °C for an hour, and subsequently stained with DAPI at 37 °C for 10 min to recognize the nuclei. Images were attained using a CLSM (OLYMPUS FV10i, Tokyo, Japan).

### 2.10. Western Blot

AGS (8 × 10^5^) cells were seeded in 6 cm plates. After overnight incubation, the cells were treated with different miR-125 (100 nM) and/or Afa (300 nM)-loaded formulations. After 24 h of incubation, the cells were lysed. The proteins were quantified using BCA protein assay (Thermo Fisher, Waltham, MA, USA). Protein (30 µg) in the volume of 10 µL was loaded for each band, separated via 10% SDS polyacrylamide gel electrophoresis (SDS-PAGE), and shifted onto PVDF membranes (Bio-Rad, Hercules, CA, USA). The membranes were blocked and incubated with primary antibodies from Cell Signaling (USA) or Abcam (UK) overnight at 4 °C. After conjugating with horseradish peroxidase (HRP)-conjugated Ig G (Jackson), the membranes were monitored using an enhanced chemiluminescence detection kit (Millipore, Billerica, MA, USA). Names and concentrations of antibodies have been demonstrated in Table S1.

### 2.11. Migration Analysis

AGS (7 × 10^4^) cells were seeded in culture insert (Ibidi, Munich, Germany) and treated with various formulations for 15 h. The images were taken through optical microscopy before and after the treatments. The migration area was quantified by ImageJ and calculated using the following equation:

Relative migration area (% of area at 0 h) = 100% − [blank area_(15h)_/blank area_(0h)_ × 100%] (3)

### 2.12. Measurement of Glutamate, Glucose Uptake, and Lipid Accumulation

AGS (1 × 10^6^) cells were seeded in 12-well plates. After treatment, the cells were incubated with Glutamate Colorimetric Assay Kit (Cayman, Ann Arbor, MI, USA), 2-NBDG (a fluorescent glucose kit; Cayman), or BODIPY™ 493/503 (a fluorescent lipid kit; Cayman), respectively, in the dark. The relative levels of glutamate, glucose uptake, and lipid accumulation were measured using a TECAN ELISA reader (Männedorf, Switzerland) at 450 nm and FACSCalibur™ flow cytometer at Ex/Em = 485/535 nm and 493/503 nm, respectively.

### 2.13. Seahorse Assay

AGS (1 × 10^6^) cells were seeded in 6-well plates for 24 h. After 24 h treatment with various formulations, the cells were transferred into seahorse plate overnight. At one hour before detection, the medium in seahorse plate was replaced by RPMI with oligomycin (ATP synthase inhibitor; Cayman), carbonylcyanide p-trifluoromethoxyphenylhydrazone (FCCP; protonophoric uncoupler; Cayman), and antimycin A (electron transport chain inhibitor; Cayman). Oxygen consumption rate (OCR) and extracellular acidification rate (ECAR) were detected using a Seahorse XF24 Extracellular Flux Analyzer (Seahorse Biosciences, North Billerica, MA, USA).

### 2.14. Measurement of Acetyl-CoA, Lactate, and ATP

AGS (1 × 10^6^) cells were seeded in 12-well plates. After 24 h treatment with various formulations, the cells were incubated with A-CoA (Acetyl Coenzyme A) ELISA Kit (Elabscience, Houston, TX, USA), D-Lactic Acid/Lactate Colorimetric Assay Kit (Elabscience), and ATP Detection Assay Kit-Luminescence (Cayman), separately. The relative luminescence levels of ATP and the relative absorbance levels of acetyl-CoA and lactate were detected using a TECAN ELISA reader at luminescence or absorbance wavelength of 450 nm and 530 nm, respectively.

### 2.15. Measurement of Mitochondrial Membrane Potential

AGS (1 × 10^6^) cells were seeded in 12-well plates. After 24 h treatment, the cells were stained with mitochondrial membrane potential detection kit JC-1 (Cayman) for 30 min. The relative level of mitochondrial membrane potential as the ratio of total fluorescence (red JC-1 aggregates: green monomer) was measured by flow cytometry at Ex/Em = 535/590 nm and 485/535 nm, respectively.

### 2.16. Measurement of Mitochondrial ROS

AGS (1 × 10^6^) cells were seeded in 12-well plates. After 24 h treatment, the cells were stained with fluorescent mitochondrial ROS kit mitoSOX™ (Thermo Fisher) for 10 min in the dark. The relative level of mitochondrial ROS was measured using FACSCalibur flow cytometer at Ex/Em = 504/529 nm.

### 2.17. Observation of Mitophagy and Mitochondrial Morphology

AGS (8 × 10^5^) cells were seeded in 6 cm plates. After 24 h treatment, the mitochondria and the nuclei were stained with MitoTracker^®^ Red for 30 min and DAPI (blue) for 10 min at 37 °C. The cells were then fixed with 4% paraformaldehyde at 37 °C for 10 min. After FBS-blocking, microtubule-associated protein 1 light chain 3 (LC3) II was labeled with primary antibody of LC3II (GeneTex, Taiwan) at 4 °C overnight and then labeled with Cy5-Ig G (Jackson, PA, USA) at 4 °C for 60 min. Images were collected using an OLYMPUS CLSM.

### 2.18. Measurement of Apoptosis

AGS (1 × 10^5^) cells were incubated in each well of 24-well plates. After 24 h of treatment, the cells were stained with Annexin V-FITC/propidium iodide (PI) Apoptosis detection kit (Strong Biotech Corporation, Taipei, Taiwan) for 20 min at 25 °C in the dark. In early apoptotic cells, phosphatidylserine is exposed in intact cell membranes and is associated with annexin V-FITC as displayed in FITC^+^/PI^-^ quadrant. Necrotic or late apoptotic cells are frequently found in FITC^+^/PI^+^ quadrant. The relative percentages of viable, apoptotic, and necrotic cells were detected and computed by flow cytometry.

### 2.19. Establishment of AGS-Bearing Mouse Model

Male BALB/c nude mice (4 to 6 weeks old) were purchased from National Laboratory Animal Center. The animal experiments were carried out under guidelines approved by the Institutional Animal Care and Use Committee (IACUC) of National Yang Ming Chiao Tung University (approval number: 1100906; approval date: 13 September 2021). AGS (5 × 10^6^ cells in 0.2 mL PBS) were injected subcutaneously into the right flank of mice to establish AGS-bearing BALB/c nude mice.

### 2.20. Antitumor Efficacy

The treatment began when the tumors grow to approximately 60 mm^3^. Tumor-bearing mice were randomly divided into seven groups (n  =  5). The therapy regimen included saline solution (CTR), miR-125/SLN-KL, Afa, Afa/SLN, Afa/SLN-KL, miR-125/SLN-KL + Afa/SLN-KL, and miR-125 + Afa/SLN-KL. Mice were administered twice a week via tail vein injection with seven formulations at equivalent Afa dose of 5 mg/kg and miR-125 dose of 1.25 mg/kg for 14 days. Tumor size and body weight were checked during the 14 days by using a digital caliper and an electronic balance. The volume (V) was calculated according to the formula:

V = (L × W^2^)/2 (4)

where length (L, mm) is the longest diameter and width (W, mm) is the shortest diameter perpendicular to the length axis.

### 2.21. Positron Emission Tomography/Computed Tomography (PET/CT)

Mice were injected by tail vein with 0.282 mCi [^18^F]-fluorodeoxyglucose (^18^F-FDG) and tumor images were visualized via PET/CT on day 15 after treatment. PET/CT images were monitored for 3 min (FOV = 80 mm) for each animal at 30 min after ^18^F-FDG injection using a LabPET/X-SPECT/X-O CT imaging system (TriFoil Imaging, Inc., Chatsworth, CA USA). The anatomical information and attenuation map for each animal were established for further image retrieval. PET and CT images were deciphered and analyzed using AMIDE software (SourceForge, IA, USA).

### 2.22. Biochemical Tests

Blood were sampled from the eye orbit of the mice after 48 h of the final treatment, and then centrifuged at 1500 rpm for 20 min. Serum was harvested for evaluation of hepatic function (glutamate pyruvate transaminase, GPT), renal function (blood urea nitrogen, BUN), cardiac function (creatine kinase-MB, CK-MB), blood glucose (GLU), cholesterol (CHO), and triacylglycerol (TG) by using the respective activity assay kits (Fujifilm, Tokyo, Japan) and a clinical dry chemistry analyzer (Fuji Dri-Chem 7000 V, Fujifilm Corp., Tokyo, Japan).

### 2.23. Terminal Deoxynucleotidyl Transferase-Mediated dUTP Nick End Labeling (TUNEL) Assay and Hematoxylin and Eosin (H&E) Staining

The mice were sacrificed after the blood collection. The tumor and tissue samples were obtained and fixed in 10% formalin. The tissues were embedded in paraffin, cut into 5 μm thick sections, and labeled with H&E. Tumor sections were deparaffinized and stained with fluorescent TUNEL kit (In Situ Cell Death Detection Kit, Roche) according to the manufacturer’s protocol. The images of TUNEL assay were visualized by fluorescence microscopy and the histology images of H&E staining were captured using an Olympus IX70 microscope (Center Valley, PA, USA).

### 2.24. Biodistribution

The tissues were frozen immediately in liquid nitrogen and then stored at −80 °C. To extract Afa, the frozen tissues were sliced into the weight of 100–200 mg and homogenize with methanol and water. After standing in ice for 15 min, the mixture was centrifuged at 3000 rpm for 15 min at 4 °C. Then, the dispersion was separated into an upper layer containing Afa, which was transferred into glass vials. The amount of Afa was detected using an Ultrospec 8000 PC spectrophotometer (Biochrom).

### 2.25. Statistical Analysis

Data are displayed as the means ± standard deviation (SD). Student’s *t*-test was performed to monitor the differences between the two treatment groups. * *p* < 0.05, ** *p* < 0.01, and *** *p* < 0.001 were defined as statistically significant differences between two groups.

## 3. Results and Discussion

### 3.1. Physicochemical Characterization of Different miR-125 and/or Afa Formulations

The conjugation between DSPE-PEG and K or L peptide was confirmed by the mass spectra of DSPE-PEG-K and DSPE-PEG-L through MALDI-TOF analysis (Figure 1B). The particle sizes of miR-125/SLN-KL, Afa/SLN-KL, and miR-125 + Afa/SLN-KL were all between 156.07 ± 1.76 and 177.87 ± 6.02 nm (Figure 1C and Table 1), which were within the optimal size range for cellular uptake, tumor permeability, and circulation times of nanoscale therapeutics [27]. These nanoscale delivery systems all exhibited positive charges of more than 30 mV. After the negatively charged miR-125 was added, miR-125 + Afa/SLN-KL became less cationic than Afa/SLN-KL. The cationic charges of the carriers were beneficial for interacting with the negatively charged membranes to enhance tumor penetration [28]. Anionic miRNAs were well protected from degradation by RNase via electrostatic interaction with cationic nanoparticles [29]. MiR-125/SLN-KL, Afa/SLN-KL, and miR-125 + Afa/SLN-KL also had polydispersity indices (PDIs) of less than 0.24 ± 0.03, indicating homogenous distribution of these three nanocarriers (Table 1). The TEM images of miR-125 and/or Afa in SLN-KL further showed spherical shape with surface shell and no aggregates (Figure 1D). As indicated by Afa’s hydrophobic characteristics, Afa may be stably inserted into the lipophilic core of SLN-KL. However, anionic miR-125 may be coated onto the cationic shell. The schematic of the distribution of miR-125 and/or Afa loaded in SLN-KL is displayed in Figure 1A. The encapsulation efficiency (EE%) and drug loading (DL%) of Afa- and/or miR-125/SLN-KL are demonstrated in Table 1. Interestingly, miR-125 was released more from miR-125 + Afa/SLN-KL than from miR-125/SLN-KL (Figure 1E, left), whereas Afa was released more from Afa/SLN-KL than from miR-125 + Afa/SLN-KL, as shown in Figure 1E (middle). Meanwhile, miR-125 was released more and faster than Afa (Figure 1E, right), reflecting the hydrophobic nature of Afa. Accordingly, miR-125 was coated onto the shell by electrostatic interaction, whereas Afa was steadily inserted into the lipid core (Figure 1D).

### 3.2. Cytotoxicity, Cellular Uptake, Transfection Efficacy, and Intracellular Trafficking of Different miR-125 and/or Afa Formulations

**Figure 2:**
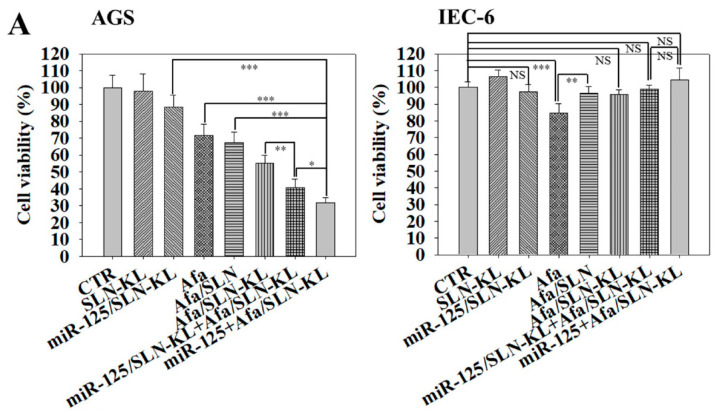

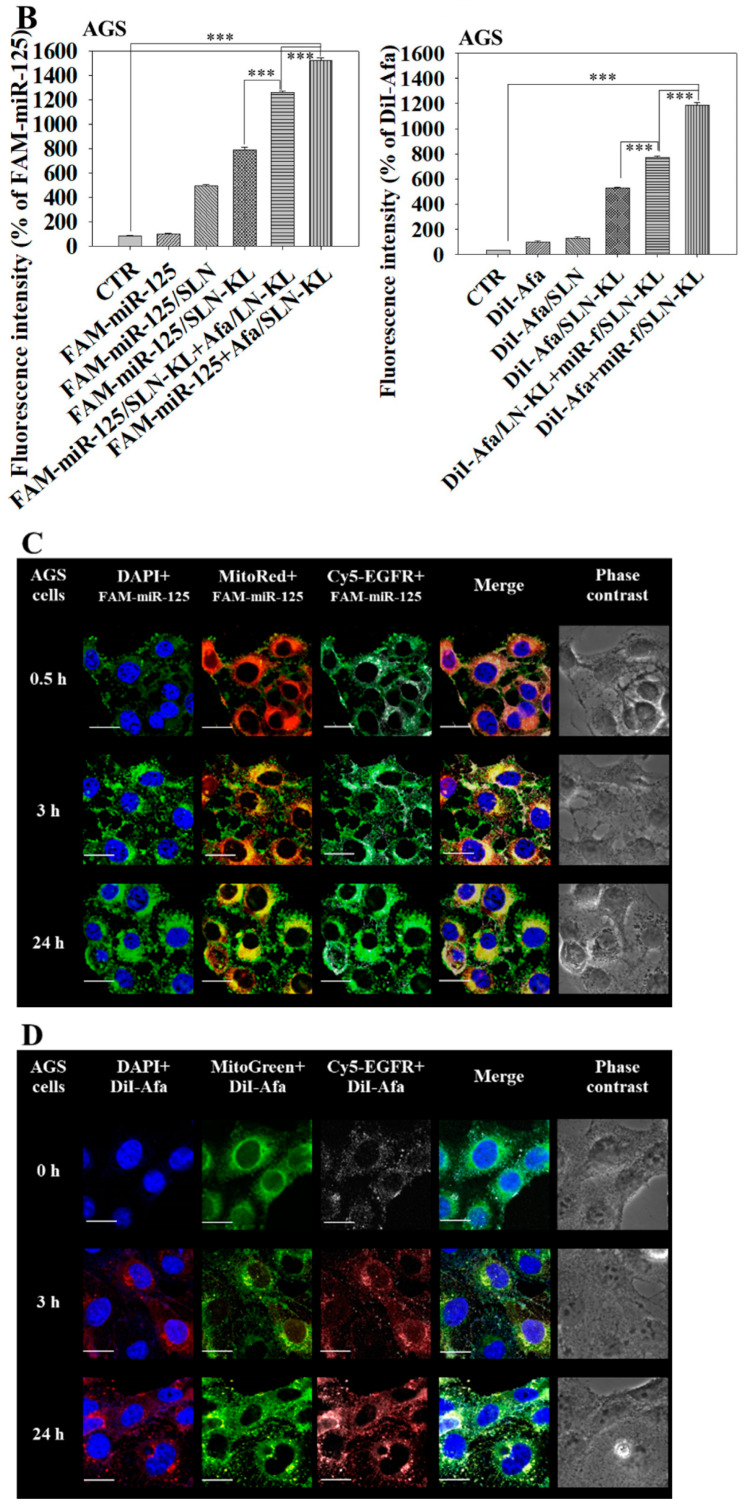
Cytotoxicity, cellular uptake, and intracellular trafficking of different Afa and/or miR-125 formulations. (**A**) Cytotoxicity of Afa and/or miR-125 formulations on (**left**) AGS cells and (**right**) IEC-6 cells for 48 h at IC30 dose of Afa (300 nM). Cell viability was determined by sulforhodamine B (SRB) assay (NS, not significant; statistical significance at * *p <* 0.05; ** *p <* 0.01; *** *p <* 0.001). (**B**) Measurement of intracellular intensity of (**left**) FAM-miR-125 (100 nM) and (**right**) DiI-Afa (100 ng/mL) in various formulations for 24 h in AGS cells by flow cytometry. (**C**) Intracellular trafficking of FAM-miR-125 + Afa/SLN-KL in AGS cells for 0/0.5, 3, and 24 h. FAM-miR-125: 100 nM; Afa: 300 nM. Blue: DAPI (a nuclear dye); red: MitoRed (a mitochondrial dye); green: FAM-miR-125 (a fluorescent miR-125); gray: Cy5-Anti-EGFR. Scale Bar, 20 µm. (**D**) Intracellular trafficking of miR-125 + DiI/SLN-KL in AGS cells for 3, 8, and 24 h. MiR-125: 100 nM; DiI: 100 ng/mL. Blue: DAPI (a nuclear dye); green: MitoGreen (a mitochondrial dye); red: DiI (a probe of Afa); gray: Cy5-Anti-EGFR. Scale bar, 20 µm.

The cytotoxicity of miR-125 and/or Afa in various formulations on GC AGS cells and non-transformed IEC-6 cells was investigated. SLN-KL did not produce noticeable toxicity to AGS and IEC-6 cells (Figure 2A). Importantly, Afa at an IC_30_ dose in the combined formulation of miR-125 + Afa/SLN-KL exerted a synergistic cytotoxic effect against AGS cells compared with the corresponding single therapy of miR-125/SLN-KL or Afa/SLN-KL (*p <* 0.001; Figure 2A, left). Strikingly, the cotreatment of miR-125 + Afa in a single nano-formulation of SLN-KL (miR-125 + Afa/SLN-KL) caused more reduction in AGS cell viability than the combination of miR-125/SLN-KL and Afa/SLN-KL in two nano-formulations (*p <* 0.05; Figure 2A, left). Figure 2A (right) shows that Afa caused a mild cytotoxicity of approximately 20% on normal IEC-6 cells. After Afa was packed into SLN or SLN-KL, either alone or in combination with miR-125, cell viability returned to approximately 100%, indicating that various SLN-based formulations may diminish the cytotoxicity of Afa on IEC-6 cell (*p <* 0.01; Figure 2A, right). Based on the cell-viability results, the synergy quantification for the combination index (CI) of cotreatment of miR-125 and Afa was calculated as 0.41 by using the Chou–Talalay method by CompuSyn software [30]. A CI level of 0.3–0.7 corresponded with a synergistic effect of miR-125 and Afa in SLN-KL.

The cellular uptake of miR-125 and Afa were detected in AGS cells by flow cytometry (Figure 2B). The uptake of FAM-miR-125, a fluorescent miR-125, was found to increase in the FAM-miR-125/SLN-KL group (Figure 2B, left). A highly significant amplification in fluorescence intensity was found in cells treated with FAM-miR-125 + Afa/SLN-KL compared with that in cells treated with FAM-miR-125 (*p <* 0.001; Figure 2B, left). Similarly, 1,10-dioctadecyl-3,3,30,30-tetramethylindocarbocyanine perchlorate (DiI) was used to label Afa in a cellular uptake study (Figure 2B, right). Compared with free DiI-Afa, the DiI-Afa/SLN-KL group displayed higher fluorescence intensity, and the most remarkable increase in fluorescence intensity was found in the single formulation of miR-125 + DiI-Afa/SLN-KL, even greater than that of miR-125/SLN-KL + DiI-Afa/SLN-KL in separate formulations (*p <* 0.05; Figure 2B, right).

FAM-miR-125 + Afa/SLN-KL (for FAM-miR-125 observation) and miR-125 + DiI-Afa-Afa/SLN-KL (for Afa observation) were then used to detect the intracellular distribution of miR-125 + Afa/SLN-KL by using CLSM (Figure 2C,D). FAM-miR-125 was initially localized in EGFR and cytoplasm at 0.5 h, but it primarily accumulated in the cytoplasm and mitochondria after 3 h, particularly at 24 h (Figure 2C). Similarly, DiI-Afa constantly accumulated in EGFR at 3 h and moved to the mitochondria and cytoplasm at 24 h (Figure 2D). The distribution pattern of miR-125 and Afa suggested the enhanced localization of the delivered Afa and miR by SLN-KL into the mitochondria and cytosol to activate the subsequent multiple signaling pathways.

### 3.3. Effects of Various miR-125 and/or Afa Formulations on ErbBs/PI3K/mTOR, Kras/Erk, and Epithelial–Mesenchymal Transition (EMT) Pathways

**Figure 3:**
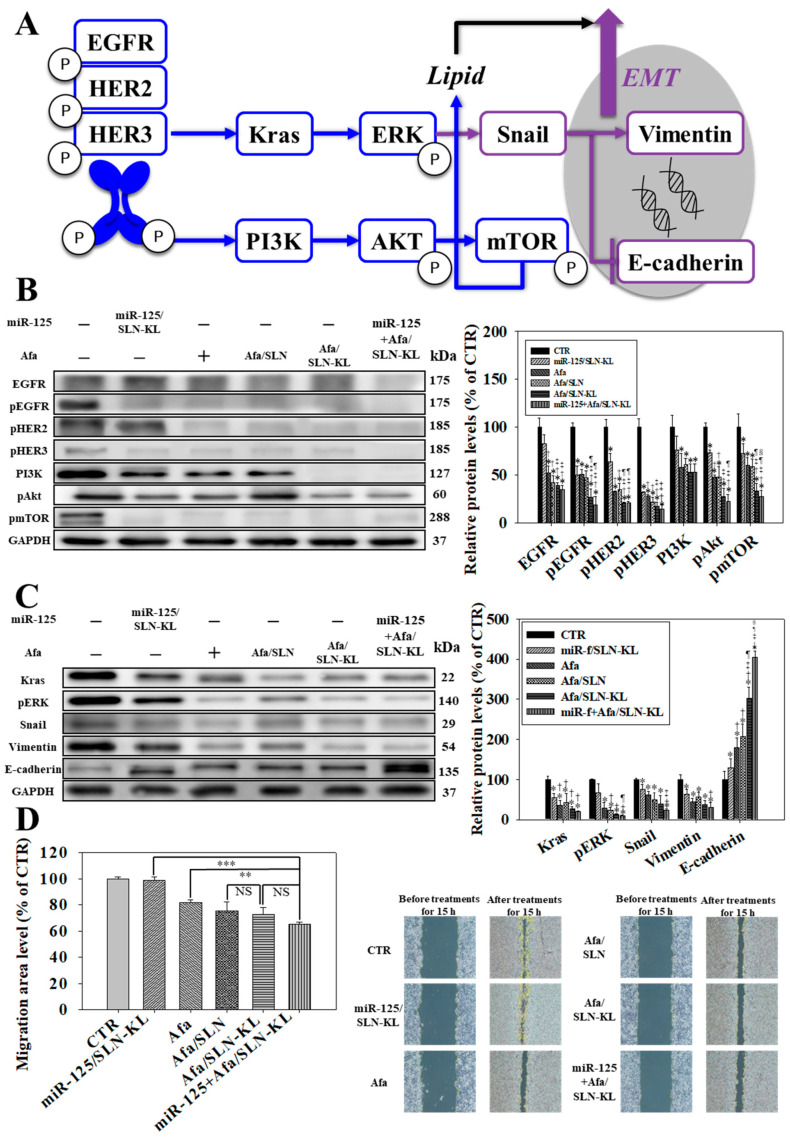
Effects of various miR-125 and/or Afa formulations on EGFR pathway in AGS cells. (**A**) Scheme of EGFR pathway. (**B**) (**Left**) Effect of various formulations of miR-125 (100 nM) and/or Afa (IC_30_: 300 nM) on expression of PI3K/Akt/mTOR pathway after treatment for 24 h on AGS cells. (**Right**) Quantification of relative protein levels of PI3K/Akt/mTOR pathway. (**C**) (**Left**) Effect of various formulations of miR-125 (100 nM) and/or Afa (IC_30_: 300 nM) on expression of Kras/Erk pathway after treatment for 24 h on AGS cells. (**Right**) Quantification of the relative protein levels of Kras/Erk pathway. (**B**,**C**) * *p* < 0.05 compared with control (CTR), ^†^ *p* < 0.05 compared with miR-125/SLN-KL, ^‡^ *p* < 0.05 compared with Afa, ^¶^ *p* < 0.05 compared with Afa/SLN, and ^§^ *p* < 0.05 compared with Afa/SLN-KL by using Student’s *t*-test analysis. (**D**) (Right) Migration assay after treatment of various formulations for 15 h. (Left) Quantification of relative percentages of cell-migration area. Migration area (% of area at 0 h) = 100% − (Blank area (15 h)/Blank area (0 h) × 100% (NS, not significant; statistical significance at ** *p <* 0.01; *** *p <* 0.001).

The effect of Afa, an irreversible pan-HER TKI [12], on overall EGFR-pathway inhibition was investigated. PI3K/Akt/mTOR and Kras/Erk pathways, the main downstream pathways of HERs, play essential roles in tumorigenesis, progression, and drug resistance [31] by causing reprogrammed metabolism and dysfunctional mitochondria in cancer cells [32]. A scheme of EGFR and EMT pathways is displayed in Figure 3A. Western blots and quantified protein levels of various formulations on the PI3K/Akt/mTOR pathway are shown in Figure 3B. HER1, HER2, and HER3 were frequently overexpressed in GC, thereby activating the downstream Kras and PI3K pathways [33]. Owing to the synergistic effect of miR-125 and Afa, PI3K, and pAkt were significantly suppressed following the inhibition of pHER1, pHER2, and pHER3. In particular, mTOR is an upstream modulator pivotal in regulating various pathways, including autophagy, anaerobic glycolysis, and fatty acid synthesis. mTOR is considered as the main target for metabolism-based anticancer therapy [34]. Strikingly, pmTOR and Kras/Erk were remarkably reduced by miR-125 + Afa/SLN-KL. Kras and pErk typically trigger Snail, subsequently inducing Vimentin and decreasing E-cadherin expression to enhance the EMT pathway [35] and thus reinforce invasion and proliferation [36]. Consequently, cancer-cell proliferation and survival in low-nutrient microenvironment are promoted [37]. Western blots and quantified protein levels of various formulations on the EMT pathway are shown in Figure 3C. Kras, pErk, Snail, and Vimentin were significantly inhibited, and E-cadherin was remarkably induced after treatment with miR-125 and/or Afa-loaded formulations, especially effective after the cotreatment of miR-125 + Afa/SLN-KL (Figure 3C). This result indicated that the EMT pathway was suppressed after the upstream inhibition of ErbBs/PI3K/mTOR and Kras/Erk pathways. Accordingly, the best inhibitory effect on cancer cell metastasis was displayed in the miR-125 + Afa/SLN-KL group, as supported by the migration assay findings of AGS cells in culture inserts (Figure 3D).

### 3.4. Effects of Various miR-125 and/or Afa Formulations on Aerobic and Anaerobic Glycolysis Pathways

**Figure 4:**
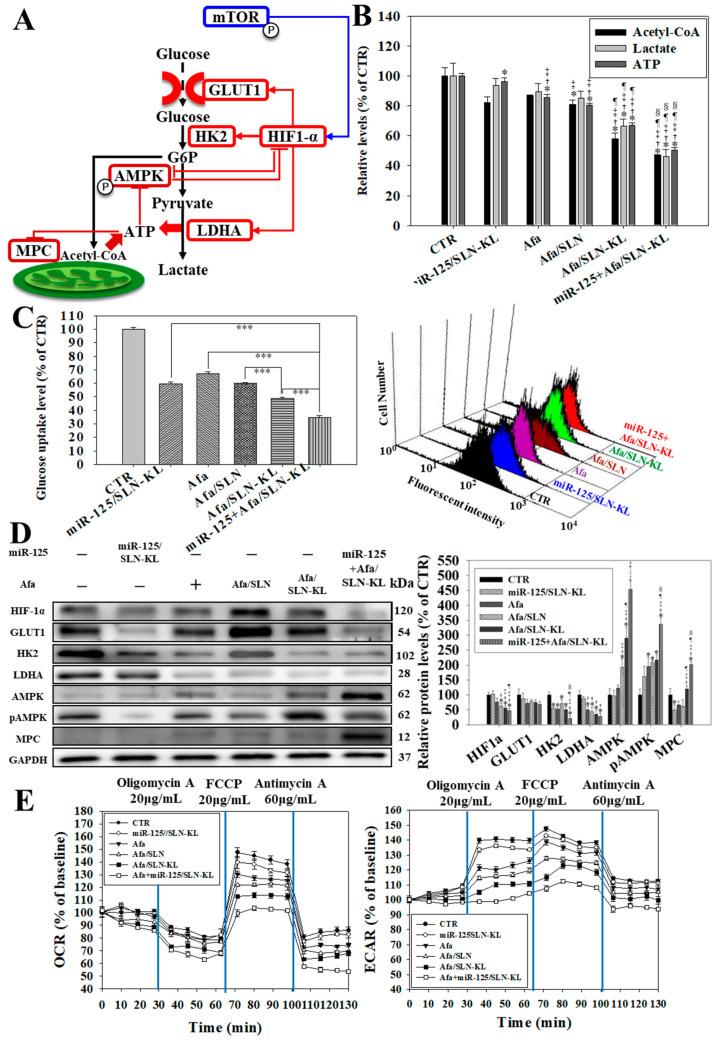
Effects of miR-125 (100 nM)- and/or Afa (300 nM)-loaded formulations on glycolysis pathway-related factors in AGS cells after 24 h treatment. (**A**) Scheme of glycolysis pathway. (**B**) Measurement of relative levels of acetyl-CoA, lactate, and ATP by using a multifunctional microplate reader. (**C**) (**Left**) Measurement of glucose uptake by detecting fluorescent analog of glucose (2-NBDG, 2-deoxy-2-[(7-nitro-2,1,3-benzoxadiazol-4-yl)amino]-D-glucose; 20 mM) with the use of a flow cytometer (statistical significance at *** *p <* 0.001). (**Right**) Histogram plots of fluorescent 2-NBDG after various treatments in AGS cells. (**D**) (**Left**) Effect of various formulations on the expression of proteins in glycolysis-related pathway. (**Right**) Quantification of relative protein levels in glycolysis-related pathway. (**B**,**D**) * *p* < 0.05: compared with CTR, ^†^ *p* < 0.05 compared with miR-125/SLN-KL, ^‡^ *p* < 0.05 compared with Afa, ^¶^ *p* < 0.05 compared with Afa/SLN, and ^§^ *p* < 0.05 compared with Afa/SLN-KL via Student’s *t*-test analysis. (**E**) Measurement of (**left**) oxygen consumption rate (OCR) and (**right**) extracellular acidification rate (ECAR) by using an XFe24 analyzer.

Glycolysis acts as an essential pathway for providing energy in various cells. An overall glycolysis scheme is shown in Figure 4A. Anaerobic glycolysis is regarded as the main metabolic pattern to supply energy from glucose in cancer cells [38]. Hypoxia inducible factor 1α (HIF1-α), which is induced by pmTOR, activates glucose transporter 1 (GLUT1), hexokinase 2 (HK2), and lactate dehydrogenase A (LDHA). After glucose is transported into cells, it undergoes catalyzed conversion into glucose 6-phosphate (G6P), subsequently producing pyruvate. Then, pyruvate changes into lactate, ultimately yielding ATP [39]. As ATP is lacking, phosphorylated adenosine monophosphate kinase (pAMPK) and mitochondrial pyruvate carrier (MPC), which are sensitive to a low ratio of (ATP/AMP), are induced [40]. Pyruvate then starts to massively access the mitochondria through reinforced MPC to undergo tricarboxylic acid (TCA) cycle and oxidative phosphorylation (OXPHOS) [41]. Consistently, the suppressed LDHA decreased anaerobic glycolysis, thereby leading to the exhaustion of ATP, lactate, and acetyl CoA (Figure 4B). Alternatively, cancer cells use aerobic metabolism to survive from ATP depletion [42]. Glucose uptake was the most inhibited in the miR-125 + Afa/SLN-KL group, even better than that of miR-125/SLN-KL and Afa/SLN-KL (Figure 4C). The inactivated GLUT1 and HK2 were also positively correlated with the inhibition of glucose uptake, as supported by the data of Western blots and the quantified protein levels shown in Figure 4D. Conversely, the AMPK, pAMPK, and MPC levels increased after treatment with miR-125 and/or Afa-loaded formulations (Figure 4D).

Multiple mechanisms could account for mitochondrial dysfunction. These mechanisms include reduced levels of lactate, acetyl-CoA, and ATP, depolarized membrane potential, and dramatically declined OCR and ECAR, at least partially owing to pro-apoptotic K peptide, Afa, and miR-125, particularly in the formulation of miR-125 + Afa/SLN-KL (Figure 4E). Dysfunctional mitochondria also inhibited aerobic glycolysis, especially signified by the decreased OCR and ECAR after treatment with miR-125 + Afa/SLN-KL (Figure 4E; right). As the main byproduct during ATP production in anaerobic respiration, the decreased lactate level was regarded as a sign of the suppressed anaerobic glycolysis of AGS cells caused by miR-125 and/or Afa-loaded SLN formulations. Therefore, miR-125 + Afa/SLN-KL inhibited anaerobic and aerobic glycolysis and modulated mitochondrial dysfunction in AGS cells (Figure 4).

### 3.5. Effects of Various miR-125 and/or Afa Formulations on the Pathways of Glutaminolysis and Fatty Acid Metabolism

**Figure 5:**
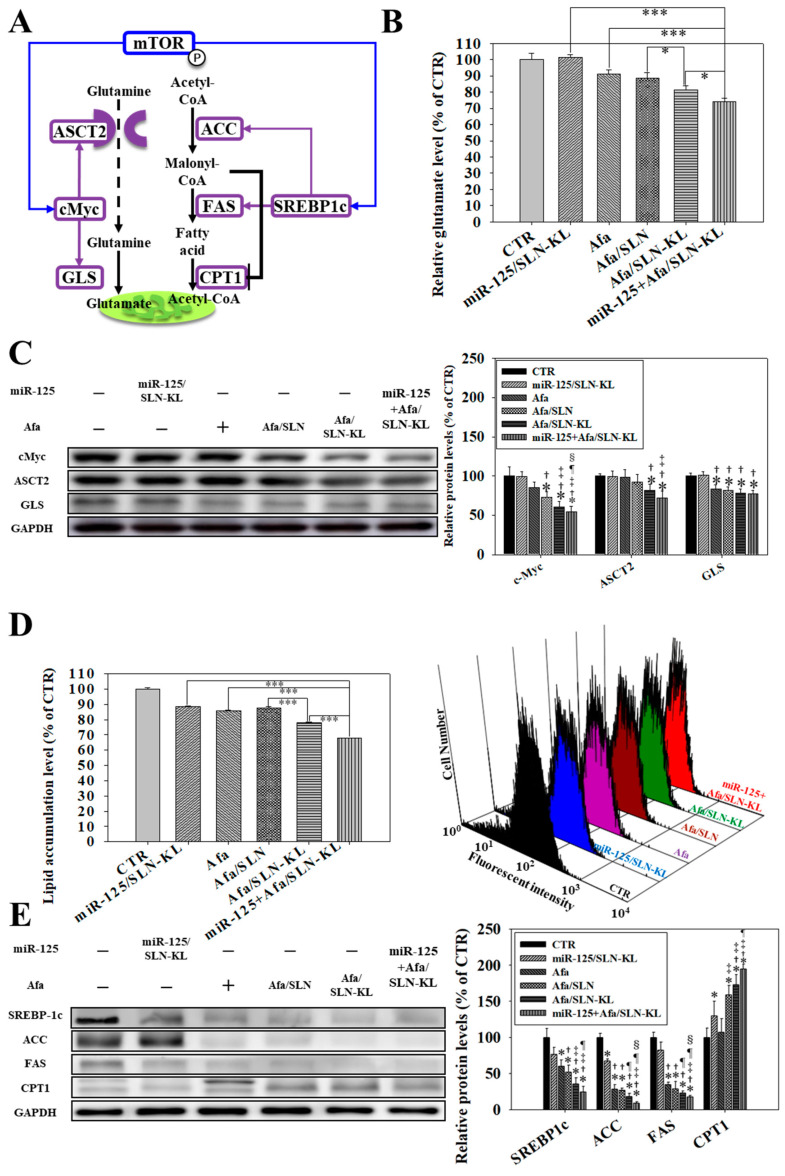
Effects of various formulations of miR-125 (100 nM) and/or Afa (300 nM) for 24 h on the pathways of glutaminolysis and fatty acid metabolism in AGS cells. (**A**) Scheme of glutaminolysis and fatty acid metabolism. (**B**) Measurement of relative glutamate levels by using a multifunctional microplate reader (statistical significance at * *p <* 0.05; *** *p <* 0.001). (**C**) (**Left**) Effect of various formulations of Afa and/or miR-125 on the expression of glutaminolysis pathway. (**Right**) Quantification of relative protein levels of glutaminolysis pathway. (**D**) (**Left**) Measurement of lipid accumulation by measuring a fluorescent lipid probe (4,4-Difluoro-1,3,5,7,8-Pentamethyl-4-Bora-3a,4a-Diaza-s-Indacene; BODIPY™; 100 mM) using a flow cytometer (statistical significance at *** *p <* 0.001). (**Right**) Histogram plots of fluorescence distribution of BODIPY™ after various treatments in AGS cells. (**E**) (**Left**) Effect of various formulations on protein expression in the pathway of fatty acid metabolism. (**Right**) Quantification of relative protein levels of fatty acid metabolism pathway. (**C**,**E**) * *p* < 0.05 compared with CTR, ^†^ *p* < 0.05 compared with miR-125/SLN-KL, ^‡^ *p* < 0.05 compared with Afa, ^¶^ *p* < 0.05 compared with Afa/SLN, and ^§^ *p* < 0.05 compared with Afa/SLN-KL via Student’s *t*-test analysis.

In addition to glucose, glutamine and fatty acid are essential energy sources [38]. After glutamine is transported into cells by using alanine serine cysteine transporter 2 (ASCT2), it is converted into glutamate in the mitochondria by glutaminase (GLS) [43]. Glutamate is metabolized to α-ketoglutarate (α-KG), and then it enters the TCA cycle to escalate ATP generation. ASCT2 and GLS are regulated by cellular-Myc (c-Myc), which is further activated by pmTOR [44]. The related scheme is displayed in Figure 5A, and it is supported by the findings in Figure 5B–E. The most profound reduction in glutamate level was found in the combined group of miR-125 + Afa/SLN-KL (Figure 5B). Moreover, Western blots and quantified protein levels demonstrated that c-Myc, ASCT2, and GLS were suppressed in the groups of Afa and/or miR-125 encapsulated in SLN-KL formulation (Figure 5C), indicating notable inhibition in mTOR and glutaminolysis by miR-125 + Afa/SLN-KL. The regulation of glutamine catabolism diminishes ATP generation and the supply of nitrogen, sulfur, and carbon skeletons for cancer-cell growth [45].

Meanwhile, fatty acids undergo intricate metabolism regulation to strike a balance between energy production and biosynthesis, thereby providing energy source for rapid proliferation of cancer cells [40]. Accordingly, fatty acid synthase (FAS) and acetyl-CoA carboxylase (ACC), which are induced by sterol regulatory element-binding transcription factor 1c (SREBP-1c) [40], participate in the transformation of malonyl-CoA into fatty acids [42]. Subsequently, fatty acids pass through carnitine palmitoyl transferase I (CPT1) on the outer membrane of the mitochondria and undergo fatty acid oxidation to produce acetyl-CoA, which then enters the TCA cycle [46]. Thus, ACC and FAS are activated to generate fatty acids and meet the demands of acetyl-CoA if SREBP-1c is induced by mTOR [47]. However, malonyl-CoA suppresses CPT1 to prevent fatty acid from accessing the mitochondria [48]. In the present study, the increased pAMPK owing to the decreased ATP inhibited SREBP-1c and pmTOR, thereby inhibiting the production of fatty acid and ATP. The schematic of this complicated mechanism is shown in Figure 5A. Consistently, the most remarkable reduction in lipid accumulation was found in the miR-125 + Afa/SLN-KL group (Figure 5D). The protein expression levels of pmTOR, SREBP-1c, ACC, and FAS were the most profoundly inhibited, and CPT1 was the most induced compared with the control (CTR) group when AGS cells were treated with miR-125 + Afa/SLN-KL (Figure 5E). Collectively, the depletion of acetyl CoA, lactate, and ATP (Figure 4B) were pivotal signals to reprogram metabolic regulation in the mitochondria by altering the utilization of glucose (Figure 4C–E), glutamine (Figure 5B,C), and fatty acid (Figure 5D,E).

### 3.6. Effects of Various miR-125 and/or Afa Formulations on Mitophagy or Mitochondrion-Mediated Apoptosis Pathway

**Figure 6:**
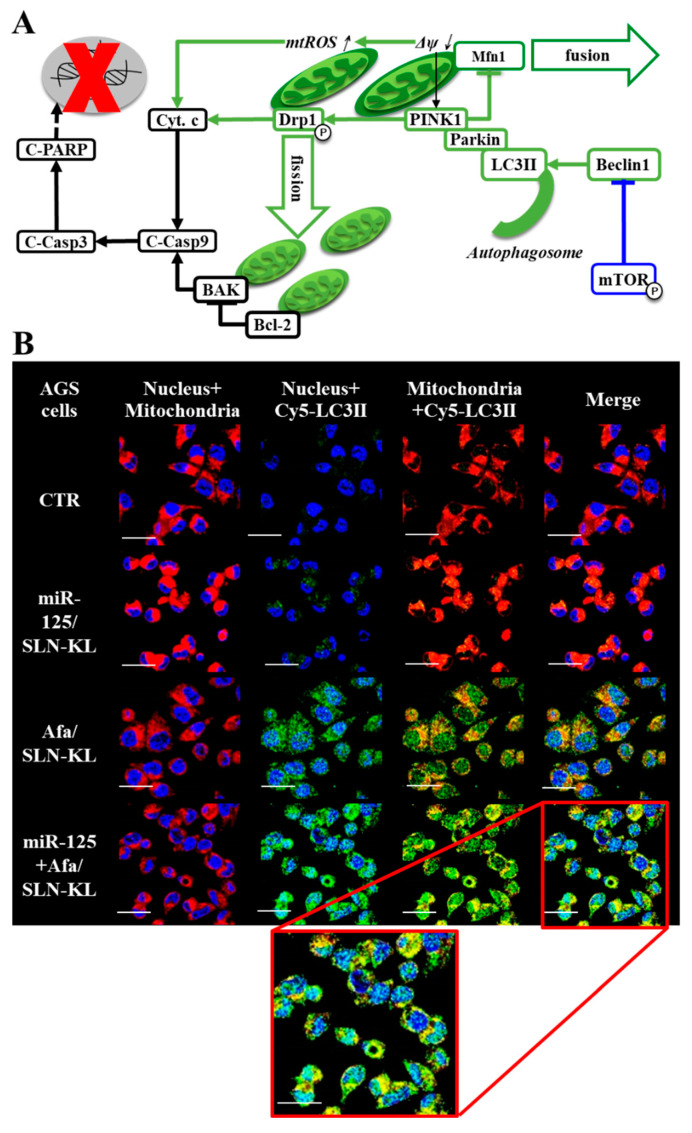

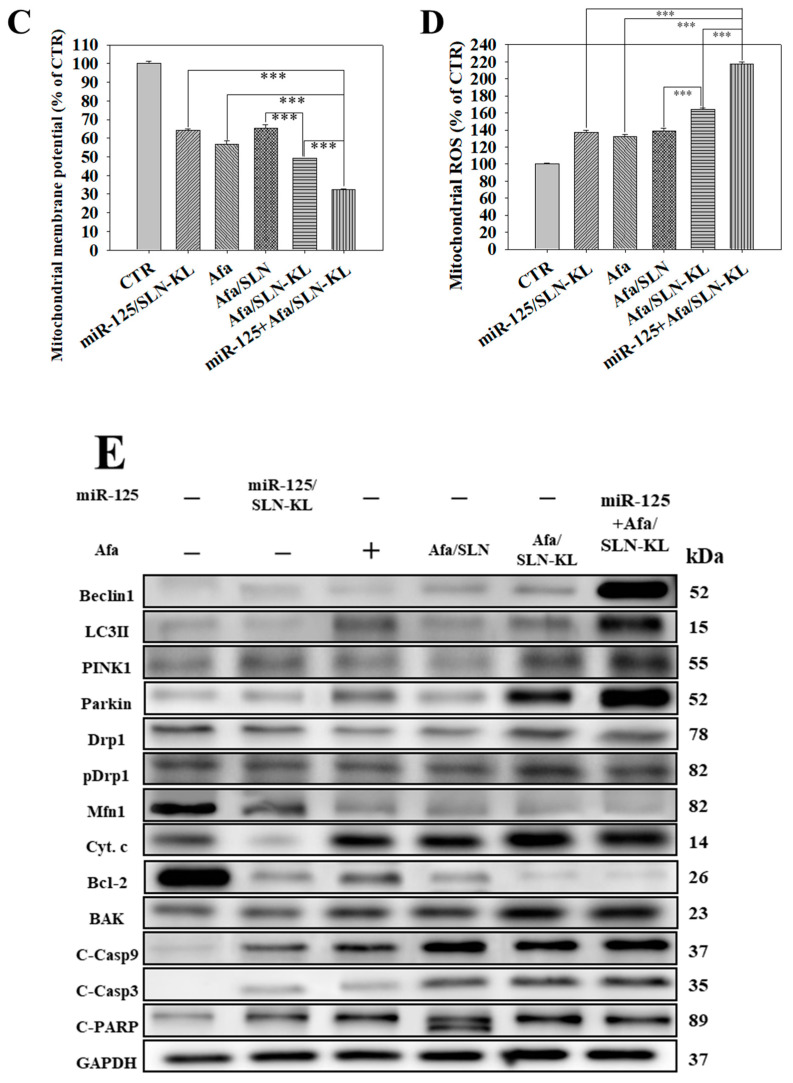

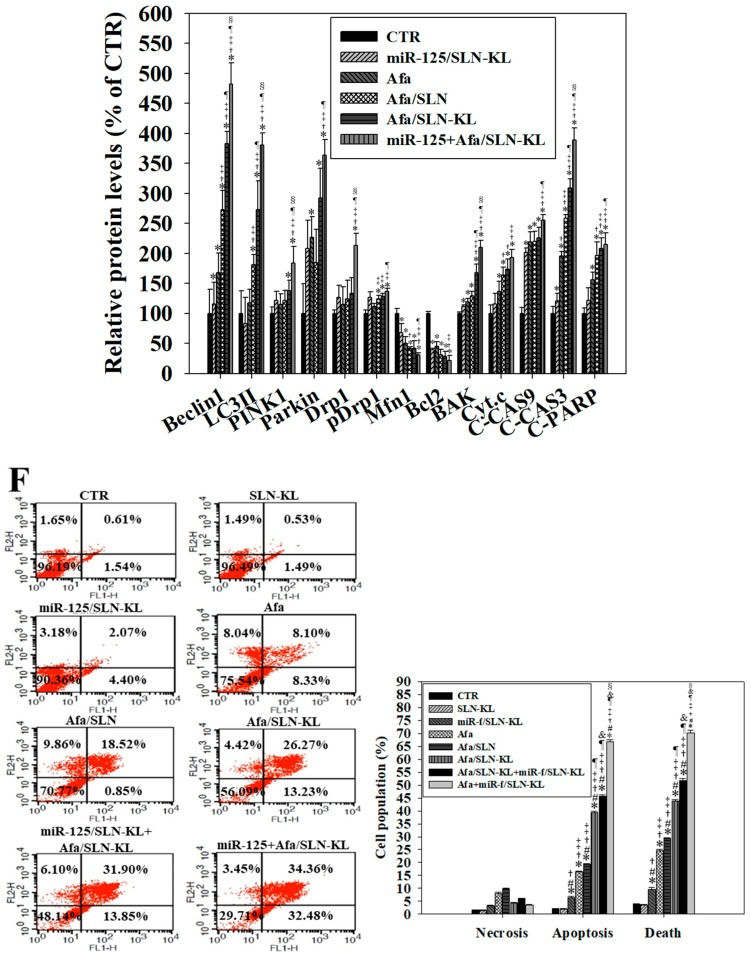
Effects of various miR-125 (100 nM) and/or Afa (300 nM) formulations on mitophagy and mitochondrion-mediated apoptosis pathway after 24 h treatment in AGS cells. (**A**) Scheme of mitophagy and mitochondrion-mediated apoptosis pathway (↑: increase; ↓: decrease). (**B**) Images of various formulations on mitophagy by using confocal laser scanning microscopy (CLSM). Blue: DAPI (a nuclear dye); red: MitoRed (a mitochondrial dye); green: Cy5-LC3II (a marker of autophagy). Scale bar, 20 µm. (**C**) Measurement of mitochondrial membrane potential (MMP; ΔΨ_m_) by using MMP kit (JC-1; 5,5′,6,6′-tetrachloro-1,1′,3,3′-tetraethyl benzimidazolo-carbocyanine iodide; 5 mg/mL) by flow cytometry (statistical significance at *** *p <* 0.001). (**D**) Measurement of mitochondrial reactive oxygen species (ROS by using a fluorescent kit (mitoSOX™) by flow cytometry (statistical significance at *** *p <* 0.001). (**E**) (**Up**) Effect of various formulations on the expression of mitophagy and mitochondrion-mediated apoptosis pathway. (**Down**) Quantification of relative protein levels of mitophagy and mitochondrion-mediated apoptosis pathway. * *p* < 0.05 compared with CTR, ^†^ *p* < 0.05 compared with miR-125/SLN-KL, ^‡^ *p* < 0.05 compared with Afa, ^¶^ *p* < 0.05 compared with Afa/SLN, and ^§^ *p* < 0.05 compared with Afa/SLN-KL via Student’s *t*-test analysis. (**F**) (**Left**) Measurement of apoptosis percentages by detecting Annexin V/PI kit with the use of a flow cytometer. (**Right**) Quantification of relative cell population percentages from Annexin V/PI assay. * *p* < 0.05 compared with CTR, # *p* < 0.05 compared with SLN-KL, ^†^ *p* < 0.05 compared with miR-125/SLN-KL, ^‡^ *p* < 0.05 compared with Afa, ^¶^ *p* < 0.05 compared with Afa/SLN, ^&^ *p* < 0.05 compared with Afa/SLN-KL, and ^§^ *p* < 0.05 compared with miR-125/SLN-KL + Afa/SLN-KL via Student’s *t*-test analysis.

Mitophagy plays a critical role in either the survival or apoptosis of cancer cells [49]. PTEN-induced kinase1 (PINK1) is pivotal in regulating mitophagy and fission to induce apoptosis [23,50]. Interestingly, dynamin-related protein 1 (Drp1)-associated mitochondrial fission and reactive oxygen species (ROS)-promoted mitochondrial damage activated mitophagy and mitochondrion-dependent apoptosis. These phenomena were evidenced by the extensive induction in fission, mitophagy, and apoptosis of AGS cells, which were triggered by miR-125- and/or Afa-loaded formulations (Figure 6). The overall scheme is shown in Figure 6A. The most widespread colocalization of LC3II and the mitochondria appeared in the miR-125 + Afa/SLN-KL group among single treatment of miR-125/SLN-KL and Afa/SLN-KL (Figure 6B). The most remarkable decrease in mitochondrial membrane potential (ΔΨ_m_) and increase in mitoROS were found in the miR-125 + Afa/SLN-KL group, leading to the greatest upregulation of cytochrome c expression (Figure 6C–E). Western blots and quantified protein levels revealed the induced Beclin1, LC3II, PINK1, and Parkin, indicating a noteworthy enhancing effect of miR-125 + Afa/SLN-KL on mitophagy (Figure 6E). The Western blot findings also supported the apoptosis induction, including the suppressed Bcl-2 and the increased expression levels of BAK, C-PARP, and C-Casp-3 and -9, especially in the case of miR-125 + Afa/SLN-KL (Figure 6E). These Afa and/or miR-125 formulations caused mitochondrial-membrane permeation to drive cytochrome c leakage into the cytosol and the increase in Casp-9 (Figure 6C,E), which also occurred in the previous study with other treatments [51]. Upon Casp-9 induction by either BAK or cytochrome c, Casp-3 and PARP were subsequently activated to induce apoptosis (Figure 6E). The greatest levels in inhibiting Bcl-2, inducing BAK, activating cytochrome c, and enhancing apoptotic cascades, occurred in the miR-125 + Afa/SLN-KL group, indicating the strongest effect of miR-125 + Afa/SLN-KL on inducing apoptosis (Figure 6E). Furthermore, miR-125 + Afa/SLN-KL prompted the most significant increases in the apoptosis and death percentages, as shown by the Annexin V/FITC assay (Figure 6F).

The depolarization of ΔΨ_m_ may trigger an increase in mitoROS [52] and an induction of PINK1 to activate Drp1 and inhibit Mitofusin-1 (Mfn1) [53]. Furthermore, mitoROS and p-Drp1 enhance cytochrome c to induce apoptosis cascade [54]. Activated Drp1 and blocked Mfn may enhance fission and suppress fusion [54]. PINK1 recruits Parkin to participate in mitophagy [49]. Furthermore, autophagy is activated when LC3II, a marker of autophagosome, and Beclin1 are triggered after pmTOR suppression [55]. Accordingly, the combined treatment of miR-125 + Afa/SLN-KL activated mitophagy/autophagy and apoptosis through the multiple modulation of PINK1/Parkin, p-Drp1/Mfn, LC3/Beclin1, and Casp/PARP, as supported by the findings in Figure 6.

### 3.7. In Vivo Antitumor Efficacy and PET/CT Imaging Studies of Different Afa and/or miR-125 Formulations

**Figure 7:**
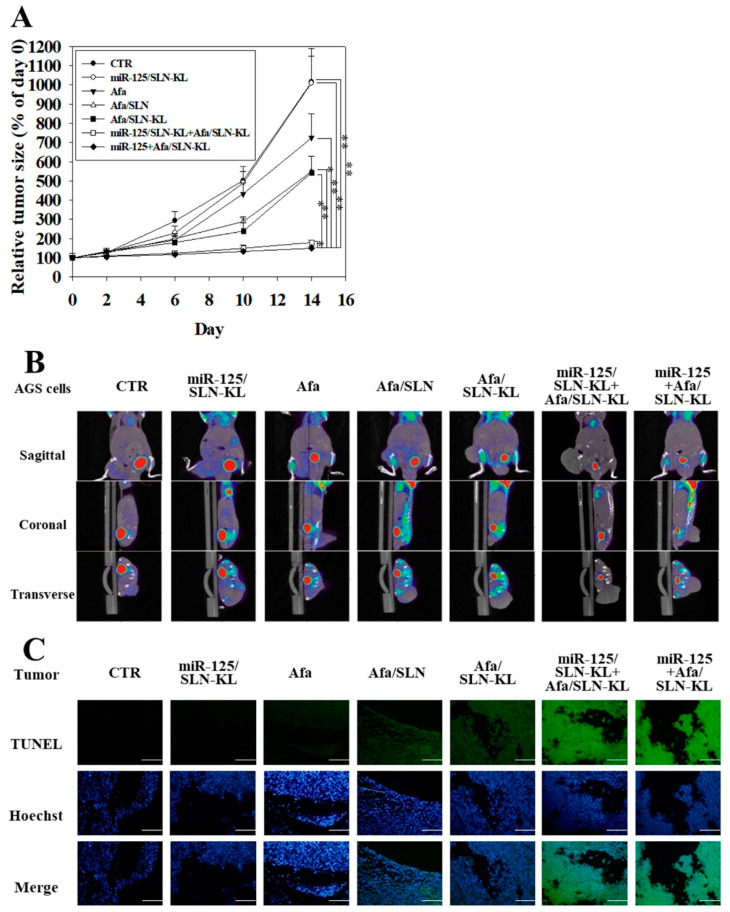
Anti-tumor efficacy of different Afa and/or miR-125 formulations on AGS-bearing mice. (**A**) Measurement of tumor volume by digital calipers every 4 days in mice treated with various formulations of Afa (5 mg/kg) and/or miR-125 (1.25 mg/kg) during 14-day therapy (statistical significance at * *p <* 0.05; ** *p <* 0.01). (**B**) PET/CT images of AGS-bearing mice by using a radiant PET/CT probe ([^18^F]-2-deoxy-2-fluoro-D-glucose; ^18^F-FDG; 0.282 mCi). (**C**) TUNEL analysis of AGS-bearing mice after completing 14-day therapy. In vivo apoptosis in tumor cells was marked (green) and the nuclei (blue) were stained with Hoechst. Scale bar, 200 µm.

The anti-cancer effect and PET/CT images of various formulations of Afa and/or miR-125 on AGS tumor-bearing BALB/c nude mice were determined. During the 14-day treatment, the tumor size was measured on days 2, 6, 10, and 14. The curve of the relative tumor size is displayed in Figure 7A. The miR-125 + Afa/SLN-KL group demonstrated better efficacy of tumor abrogation than the CTR group. The percentage in reducing tumor size was also significantly better than that in the individual group of miR-125/SLN-KL or Afa/SLN-KL, indicating the combinative enhancement in the antitumor effect of miR-125 + Afa/SLN-KL. Although Afa showed the ability to suppress tumor growth, KL peptide-modified nano-formulations displayed much more effect on antitumor efficacy. In addition, PET/CT images were obtained to concretely evaluate the alteration in tumor sizes after treatment by various formulations (Figure 7B). [^18^F]-fluorodeoxyglucose (^18^F-FDG), an analog of glucose with radiant fluorine, is extensively used as a probe in PET/CT examination to visualize tumor size. PET/CT scanning also offers results for the level of tumor suppression and the degree of reduction in glucose uptake, because glucose acts as the main energy supply of tumors [56]. The PET/CT result (Figure 7B) was similar to the tumor-size curve (Figure 7A). Moreover, among all treatment groups, miR-125 + Afa/SLN-KL had the most remarkable decrease in tumor fluorescence (Figure 7B), indicating its lowest glucose uptake level among all groups. These findings provided further evidence to support the in vitro result in inhibiting glucose uptake and reducing the energy source by using formulations of Afa and/or miR-125 (Figure 4). In vivo apoptotic analysis was further explored by TUNEL staining (Figure 7C). Apoptotic or necrotic cells of tumors were denoted as green fluorescence in TUNEL staining samples, as exhibited in Figure 7C. The brightest green fluorescence was detected in the groups of miR-125 + Afa/SLN-KL and miR-125/SLN-KL + Afa/SLN-KL compared with slight or no fluorescence in other groups, supporting the superior effect of combinative formulation on inducing in vivo apoptotic tumor cells (Figure 7C). On the basis of these findings, the best anti-tumor efficacy of the combinatorial formulation was supported by in vivo data, although only a slight difference existed between miR-125 + Afa/SLN-KL and cotreatment of miR-125/SLN-KL and Afa/SLN-KL (Figure 7A). Despite this defect, a breakthrough was achieved in simplifying drug preparation and administration by using the one-shot formulation of miR-125 + Afa/SLN-KL.

### 3.8. Biosafety Issues and Biodistribution Studies of Various Afa and/or miR-125 Formulations

**Figure 8:**
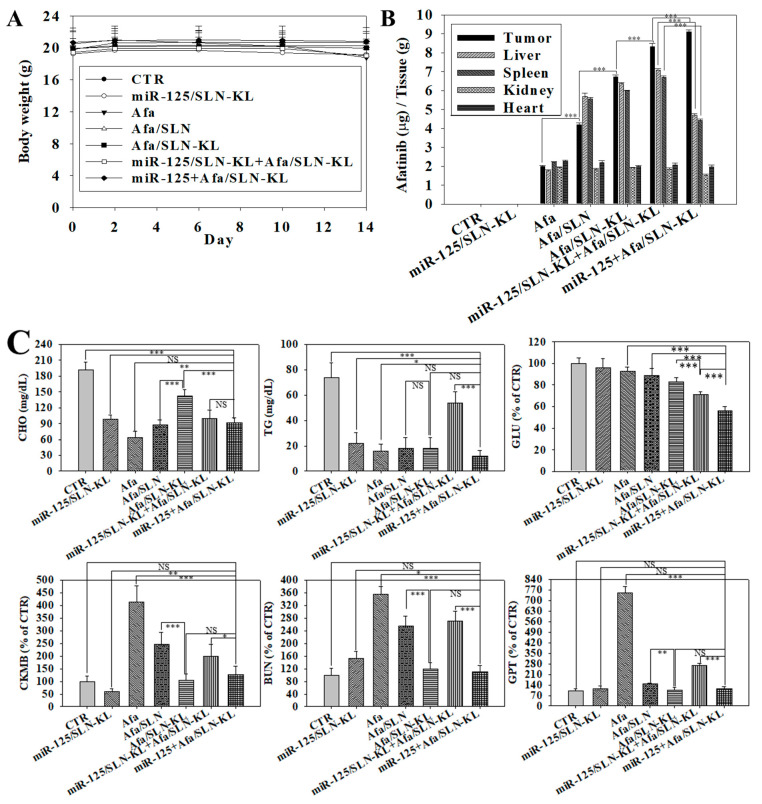

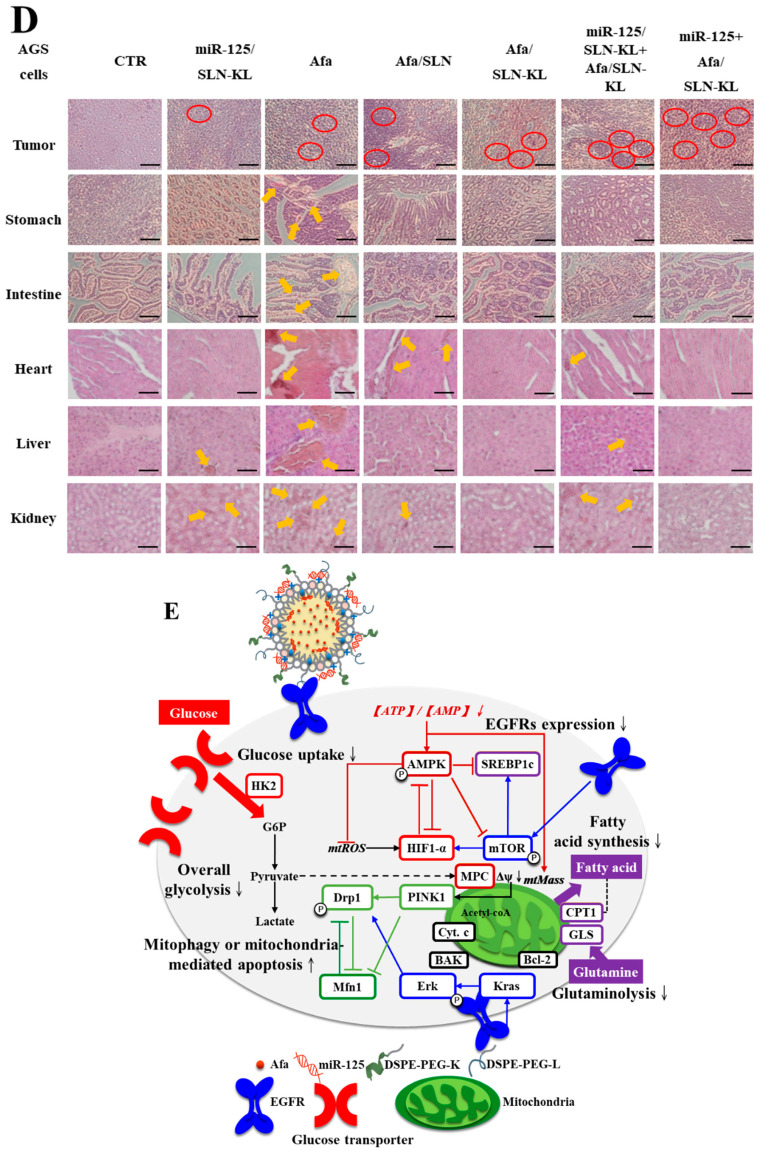
Biosafety and biodistribution studies of various formulations of Afa (5 mg/kg) and/or miR-125 (1.25 mg/kg) in AGS-bearing mice. (**A**) Measurement of body weight of AGS-bearing mice every 4 days after treatment with various formulations for 14 days. (**B**) Biodistribution study of different formulations after completion of 14-day therapy. (**C**) Blood biochemical indices of glucose (GLU), cholesterol (CHO), and triglycerides (TGs) and functions of liver by glutamic pyruvic transaminase (GPT), kidney by blood urea nitrogen (BUN), and heart by creatine kinase-MB (CK-MB) after finishing 14-day therapy. For (B, C): NS, not significant; statistical significance at * *p <* 0.05; ** *p <* 0.01; *** *p <* 0.001. (**D**) Histological photomicrographs of the tumor, stomach, kidneys, liver, heart, and intestinal sections in AGS-bearing mice after completion of 14-day therapy, as stained by H&E. Red circles indicated regions of necrosis or apoptosis, and yellow arrows denoted signs of inflammation. Scale bar, 200 μm. (**E**) Overall scheme of reprograming of dysregulated metabolism and dysfunctional mitochondria in AGS cells by miR-125 + Afa/SLN-KL (↑: increase; ↓: decrease).

No significant difference among various formulations was observed in the change of body weight (Figure 8A). The tissue distribution of Afa and/or miR-125 in various formulations was also monitored in AGS-bearing mice (Figure 8B). The biodistribution results suggested that most Afa-loaded formulations were accumulated in tumors, especially in the miR-125 + Afa/SLN-KL group. The macrophages in the spleen and liver were found to be the major routes in the reticuloendothelial system for phagocytic removal of nanoparticles [57]. In the present study, Afa and miR-125-loaded formulations were also substantially gathered in the liver and spleen (Figure 8B), owing to the activated reticuloendothelial system in these two organs to eliminate nanoparticles. Impressively, miR-125 + Afa/SLN-KL was targeted more to tumor sites and accumulated less to the liver and spleen than Afa/SLN, Afa/SLN-KL, or miR-125/SLN-KL + Afa/SLN-KL (Figure 8B; all *p* < 0.001). Cholesterol, triglycerides, and glucose were also reduced to various degrees by the single or cotreatment of Afa and/or miR-125, as indicated by biochemical indices in Figure 8C. In addition, the activity levels of glutamic pyruvic transaminase (GPT), blood urea nitrogen (BUN), and creatine kinase-MB (CK-MB) were tested to evaluate hepatotoxicity, nephrotoxicity, and cardiotoxicity, respectively. Strikingly, Afa-free drugs resulted in the highest increases of GPT, BUN, and CK-MB, indicating its toxicity to the liver, kidneys, and heart. Conversely, Afa and/or miR-125 SLN formulations reduced these toxicities in the liver, kidneys, and heart. The single formulation of miR-125 + Afa/SLN-KL decreased the organ toxicities more than the cotreatment of miR-125/SLN-KL and Afa/SLN-KL (Figure 8C, lower panels). Moreover, the histological assessment of these formulations on tumor, stomach, and intestines in AGS-bearing mice were further evaluated (Figure 8D). No signs of necrosis and apoptosis were demonstrated in the CTR group of tumor samples (Figure 8D, left in the first panels). The more intense purple area indicated a greater accumulation of apoptotic or necrotic cells in miR-125 + Afa/SLN-KL than in miR-125/SLN-KL and/or Afa/SLN-KL. The greatest amounts of apoptotic and necrotic tumor tissues (Figure 8D, first row of panels) were consistent with the TUNEL assay results (Figure 7C), suggesting that the EGFR-targeting and mitochondrion-directing peptides in SLN-KL increased tumor accumulation and an apoptosis-inducing effect. Furthermore, the yellow arrows denoted different degrees of tissue lesions in the stomach, intestines, heart, liver, and kidneys (Figure 8D, second to sixth panels). The Afa groups displayed mononuclear cell infiltration and/or inflammation in various organs, indicating the occurrence of hepatic, nephrotic, cardiac, and GI toxicities. GI-associated symptoms, such as diarrhea, occurred after Afa treatment in approximately 88% of patients [12]. MiR-125 + Afa/SLN-KL and miR-125/SLN-KL + Afa/SLN-KL treatments notably improved the drawbacks of Afa (Figure 8D, second to sixth panels). However, miR-125/SLN-KL+ Afa/SLN-KL treatment still led to a mild mononuclear cell infiltration in the heart, liver, and kidneys (Figure 8D, fourth to sixth panels). The results of anti-tumor efficacy and biosafety revealed that the one-shot administration of miR-125 + Afa/SLN-KL in a combinatorial formulation exhibited effectiveness and synergism in inhibiting tumor growth and reducing organ toxicities compared with the combined treatment of miR-125/SLN-KL and Afa/SLN-KL (Figure 7 and Figure 8). The overall scheme of the reprograming of dysregulated metabolism and dysfunctional mitochondria in AGS cells by miR-125 + Afa/SLN-KL is shown in Figure 8E.

## 4. Conclusions

A solid lipid-nanoparticle formulation of miR-125 + Afa/SLN-KL coated with mitochondrial targeting K peptide and EGFR-directed L ligand was prepared. It was successfully used to inhibit the pan-ErbB-facilitated EMT and mTOR-mediated discoordination of glycolysis–glutaminolysis–lipid metabolism for the potential treatment of GC. This cotreatment modulated numerous critical enzymes or proteins, such as the EGFR/HER2/HER3, Kras/ERK/Vimentin, and mTOR/HIF1-α/HK2/LDHA pathways. As a result of suppression of glutaminolysis, glycolysis, and mitochondrial OXPHOS and the disruption of fatty acid synthesis, miR-125 + Afa/SLN-KL triggered an obvious decline in the levels of lactate, acetyl-CoA, and ATP, thereby provoking cancer-cell apoptosis. The increased expression of Beclin1, LC3II, PINK1/Parkin, and Drp1 directed the involvement of mitophagy in supporting the apoptosis induction of GC cells, which subsequently caused the breakdown of tumor-cell homeostasis and death. In vivo findings on AGS-bearing mice revealed that miR-125 + Afa/SLN-KL had the best antitumor efficacy and safety. The combinatorial formulation disturbed the metabolic reprogramming, alleviated the “Warburg effect” of tumors, interrupted the supply of cholesterol and triglyceride, and exacerbated the energy depletion in the tumor microenvironment, thereby inhibiting tumor proliferation and aggressiveness. Collectively, the results indicated that this one-shot formulation of miR-125 + Afa/SLN-KL was a breakthrough in simplifying drug preparation and administration and effectively inhibiting tumor progression via the versatile targeting of pan-ErbB- and mTOR-mediated mitochondrial dysfunction and dysregulated metabolism. Consequently, mitophagy- and apoptosis-associated tumor regression in GC is activated.

## Figures and Tables

**Table 1:** Characterization of various formulations.

	miR-125/SLN-KL	Afa/SLN-KL	miR-125 + Afa/SLN-KL
Size (nm)	156.07 ± 1.76	170.87 ± 4.02	177.87 ± 6.02
PDI ^a^	0.22 ± 0.02	0.17 ± 0.04	0.24 ± 0.03
Zeta potential (mV)	32.20 ± 0.20	39.30 ± 2.80	35.97 ± 0.44
EE% ^b^ of Afa	--	89.03 ± 0.06	91.37 ± 0.05
EE% of miR-125	88.67 ± 0.04	--	86.71 ± 0.03
DL% ^c^ of Afa	--	17.22 ± 0.01	18.34 ± 0.01
DL% of miR-125	16.64 ± 0.06	--	14.63 ± 0.02

^a^ Polydispersity index (PDI). ^b^ Encapsulating efficiency (EE%). ^c^ Drug loading capacity (DL%).

## Data Availability

All data and materials are included in the manuscript.

## Supplementary Materials

The following supporting information can be downloaded at: www.mdpi.com/article/10.3390/pharmaceutics14091759/s1. Table S1. Antibodies used in the present study.
