# Supplementary material for: Two-in-One Nanoparticle Formulation to Deliver a Tyrosine Kinase Inhibitor and microRNA for Targeting Metabolic Reprogramming and Mitochondrial Dysfunction in Gastric Cancer
Source: Pharmaceutics. 2022 Aug 23;14(9):1759. doi: 10.3390/pharmaceutics14091759 (PMC9504622; doi:10.3390/pharmaceutics14091759)
Supplement: Supplementary file 1 [file pharmaceutics-14-01759-s001.zip › pharmaceutics-1887887-supplementary.pdf]

Table S1. Antibodies used in the present study.

Name of antibody	Company	Dilution
**Primary antibody**		
Mouse antibody anti-GLUT1	Proteintech	1:3000
Mouse antibody anti-LDHA	Proteintech	1:3000
Rabbit antibody anti-ACC	Cell Signaling Tech.	1:3000
Rabbit antibody anti-AMPK	GeneTex	1:3000
Rabbit antibody anti-BAK	GeneTex	1:3000
Rabbit antibody anti-Bcl-2	GeneTex	1:3000
Rabbit antibody anti-Beclin1	Sino	1:3000
Rabbit antibody anti-Caspase-9	Cell Signaling Tech.	1:3000
Rabbit antibody anti-Caspase-3	Cell Signaling Tech.	1:3000
Rabbit antibody anti-CPT1	ABclonal	1:3000
Rabbit antibody anti-Cyt. c	Santa Cruz	1:3000
Rabbit antibody anti-Drp1	Cell Signaling Tech.	1:3000
Rabbit antibody anti-E-cadherin	Cell Signaling Tech.	1:3000
Rabbit antibody anti-EGFR	Cell Signaling Tech.	1:3000
Rabbit antibody anti-FAS	ABclonal	1:3000
Rabbit antibody anti-GAPDH	Cell Signaling Tech.	1:3000
Rabbit antibody anti-HIF1-α	Cell Signaling Tech.	1:1000
Rabbit antibody anti-HK2	Cell Signaling Tech.	1:3000
Rabbit antibody anti-Kras	Sino	1:3000
Rabbit antibody anti-LC3II	GeneTex	1:1000
Rabbit antibody anti-Mfn1	Cell Signaling Tech.	1:3000
Rabbit antibody anti-MPC	ABclonal	1:3000
Rabbit antibody anti-Parkin	GeneTex	1:3000
Rabbit antibody anti-PARP	Cell Signaling Tech.	1:3000
Rabbit antibody anti-PINK1	BioVision	1:1000
Rabbit antibody anti-p-AKT	Cell Signaling Tech.	1:1000
Rabbit antibody anti-p-AMPK	Cell Signaling Tech.	1:3000
Rabbit antibody anti-p-Drp1	Cell Signaling Tech.	1:3000
Rabbit antibody anti-p-EGFR	Cell Signaling Tech.	1:3000

Rabbit antibody anti-p-Erk	Cell Signaling Tech.	1:3000
Rabbit antibody anti-p-HER2	Cell Signaling Tech.	1:3000
Rabbit antibody anti-p-HER3	Cell Signaling Tech.	1:3000
Rabbit antibody anti-p-mTOR	ABclonal	1:3000
Rabbit antibody anti-p-STAT3	Cell Signaling Tech.	1:3000
Rabbit antibody anti-Snail	Cell Signaling Tech.	1:3000
Rabbit antibody anti-SREBP1c	ABclonal	1:3000
Rabbit antibody anti-Vimentin	Taiclone	1:1000
**Secondary antibody**		
Peroxidase-conjugated AffiniPure Goat Anti-Rabbit IgG (H+L)	Jackson ImmunoResearch Inc.	1:1000
Peroxidase-conjugated AffiniPure Goat Anti-Mouse IgG (H+L)	Jackson ImmunoResearch Inc.	1:1000
